# Performance of ChatGPT in dental implant treatment planning: evaluation using the modified DISCERN, Global Quality Score, and accuracy–safety score

**DOI:** 10.1186/s12903-026-08009-y

**Published:** 2026-03-28

**Authors:** Aylin Ekmekcioğlu, Büşra Karaca, Afra Nur Ülgen

**Affiliations:** Department of Oral and Maxillofacial Surgery, Faculty of Dentistry, Mehmet Akif Ersoy University, Burdur, Turkey

**Keywords:** Artificial Intelligence, Diagnosis, Oral and Maxillofacial Surgery, Large Language Models

## Abstract

**Background:**

Large language models (LLMs) such as ChatGPT are increasingly used to access health-related information, including in dental implant treatment planning. However, the reliability, quality, and clinical accuracy of information provided by these AI systems remain uncertain, raising potential concerns for patient safety. This study aims to systematically evaluate ChatGPT’s performance in dental implant treatment planning, focusing on the reliability, quality, and clinical validity of its responses.

**Methods:**

Sixty clinical scenarios for dental implant treatment were designed and presented to ChatGPT. Scenarios were divided into two categories: (1) patients with deficient alveolar bone and (2) patients with systemic conditions. AI-generated responses were independently evaluated by three board-certified oral and maxillofacial surgeons. Information reliability was assessed using the Modified DISCERN instrument, overall content quality using the Global Quality Score (GQS), and clinical accuracy and safety using a Likert scale. Statistical analyses were performed with IBM SPSS Statistics 26.0. Data normality was evaluated with the Shapiro–Wilk test, and non-parametric comparisons were conducted using the Mann–Whitney U test and Spearman rank correlation. Statistical significance was set at *p* < 0.05.

**Results:**

GQS was significantly higher for systemic disease scenarios (3.83 ± 0.69) compared to bone deficiency scenarios (3.20 ± 0.40) (U = 675, *p* < 0.05). Correlation analyses revealed significant positive relationships among the evaluation scales. Specifically mDISCERN score showed a weak positive correlation with GQS (*r* = 0.325; *p* = 0.011) and a moderate positive correlation with clinical Accuracy & Safety (*r* = 0.535; *p* < 0.001). In bone deficiency scenarios, mDISCERN correlated moderately with GQS (*r* = 0.512; *p* = 0.004) and strongly with Accuracy & Safety (*r* = 0.651; *p* < 0.001), while GQS also demonstrated a strong correlation with Accuracy & Safety (*r* = 0.682; *p* < 0.001). For systemic disease scenarios, mDISCERN showed a moderate positive correlation with Accuracy & Safety (*r* = 0.473; *p* = 0.008).

**Conclusion:**

ChatGPT can be used as an adjunctive tool in dental implant planning but should not replace professional clinical judgment. Safe and effective implant management requires adherence to evidence-based guidelines and consultation with experienced specialists.

**Supplementary Information:**

The online version contains supplementary material available at 10.1186/s12903-026-08009-y.

## Background

### Introduction

Artificial intelligence (AI), particularly its deep learning subset, has progressively been integrated into healthcare systems and is beginning to exert a measurable impact across multiple medical fields, including dentistry. AI-based applications have demonstrated potential advantages such as rapid interpretation of medical images, optimization of clinical workflows, and support in reducing diagnostic and treatment-related errors [1]. In parallel, large language models (LLMs) have increasingly been explored for applications such as patient education and clinical decision support [2]. Through natural language processing, LLMs can analyze textual clinical information and generate responses that may assist healthcare professionals in understanding diagnostic and treatment-related concepts [3].

ChatGPT, a large language model developed by OpenAI, has been trained on large-scale textual data, including medical literature, and is capable of interacting with users through natural language. It has been proposed as a tool to support tasks such as answering patient inquiries, explaining treatment options, and providing general health-related information [4, 5]. However, despite these potential applications, LLMs are not without important limitations. In dentistry, and particularly in implantology, inaccurate or incomplete information may have direct implications for treatment planning and patient safety. LLM-generated responses may be affected by hallucinations, lack of access to up-to-date clinical guidelines, oversimplification of complex clinical scenarios, and sensitivity to prompt formulation, which may lead to variability in outputs and potential misinterpretation of clinical recommendations [5].

Within dentistry, dental implant therapy represents one of the most complex clinical decision-making processes, requiring the integration of anatomical, biological, and systemic factors. Dental implants are widely used in contemporary dentistry as artificial tooth roots placed within the jawbone to replace missing teeth [6]. Primary indications for dental implant therapy include single and multiple tooth loss, complete edentulism, prosthetic rehabilitation, and the restoration of esthetic and functional outcomes [7, 8]. Implant therapy is generally indicated for adult patients with sufficient bone volume and healthy periodontal tissues and may also be considered in individuals with well-controlled systemic conditions, provided that no factors are present that could adversely affect healing [9, 10]. The success of implant therapy is influenced by multiple patient- and site-related factors, including bone density, oral hygiene, smoking status, and general health status [7, 10]. Consequently, comprehensive clinical and radiographic evaluation is essential prior to implant planning, along with careful consideration of patient expectations and prosthetic requirements [6].

Given the multifactorial nature of implant-related decision-making and the increasing tendency of patients and clinicians to consult AI-based tools for health-related information, the accuracy, reliability, and safety of information generated by such systems are of particular importance. Erroneous or misleading content may compromise clinical judgment and pose potential risks to patients. Previous studies have suggested that ChatGPT and other LLMs can provide general dental and medical information; however, their performance appears to vary depending on the clinical domain and specific scenario, underscoring the need for systematic evaluation [4, 5].

Therefore, the present study aimed to systematically evaluate ChatGPT-5.2 responses to dental implant–related scenarios—particularly those involving bone insufficiency and systemic disease, which are critical determinants of implant treatment planning—with respect to reliability, quality, and accuracy/safety. In addition, response consistency and reproducibility were assessed to better characterize the strengths and limitations of LLM-based systems when applied to implant dentistry. The primary research question of this study was whether ChatGPT-5.2 can generate reliable, high-quality, and clinically accurate/safe responses to open-ended dental implant–related scenarios. In addition, the study explored the consistency and reproducibility of the model’s responses when the same clinical scenarios were presented repeatedly. Given the exploratory nature of this investigation and the limited existing evidence regarding the use of large language models in implant dentistry, no a priori directional hypotheses were formulated.

## Materials and methods

### Question Source and Study Design (Case Selection and Scenario Development)

**Fig. 1:**
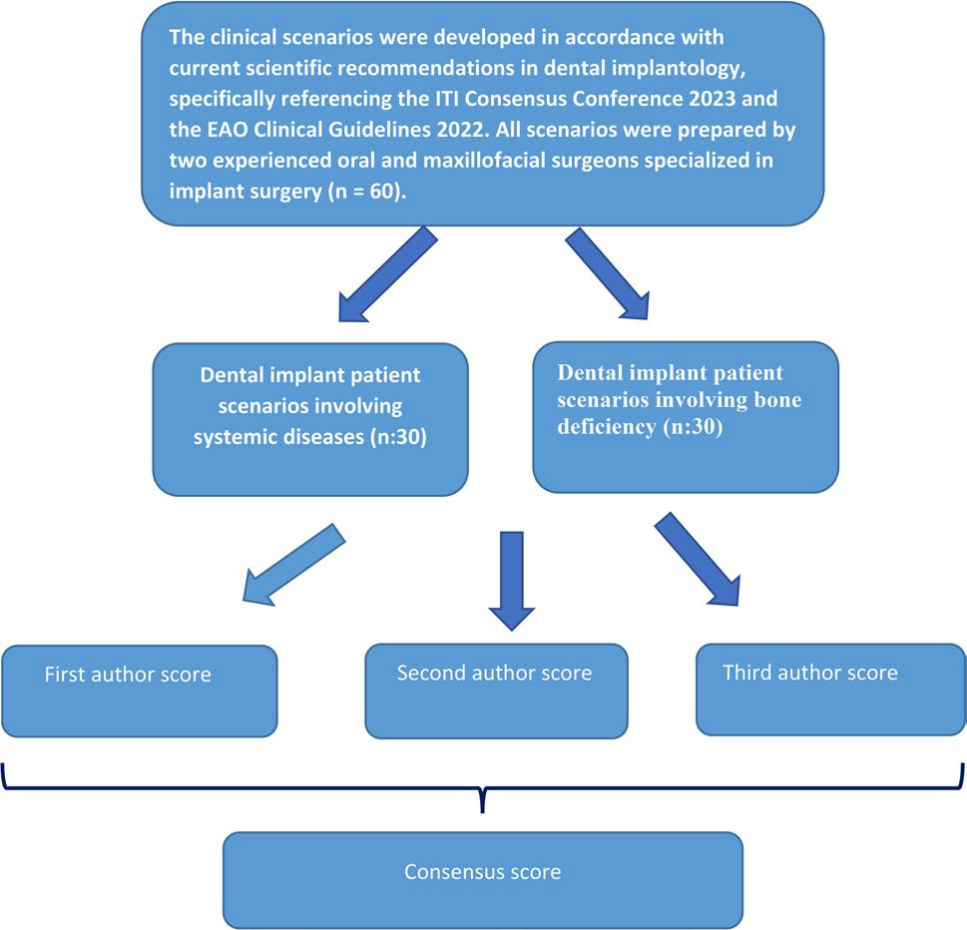
Flow chart of question selection and consensus score

In this study, 60 clinical patient scenarios regarding dental implant treatment planning were developed and categorized into two groups: patients with systemic diseases and patients with insufficient bone volume. The scenarios were purposefully constructed by two board-certified oral and maxillofacial surgeons with expertise in implantology, based on their clinical experience and current scientific recommendations. Guideline-based considerations were informed by the International Team for Implantology (ITI) 2023 Consensus Conference and the European Association for Osseointegration (EAO) 2022 Clinical Guidelines. Any discrepancies during scenario development were resolved through consultation with a third board-certified oral and maxillofacial surgeon (Fig. 1). Disagreements between the two primary surgeons primarily concerned the level of clinical detail to be included, the categorization of bone defect morphology, and the extent to which systemic conditions should influence the proposed treatment complexity. These discrepancies arose during the scenario drafting phase rather than during data analysis. The third surgeon reviewed the contested elements, particularly in cases where differing interpretations of guideline application or risk stratification were present, and facilitated resolution through structured discussion grounded in current ITI and EAO recommendations. As the objective of this phase was scenario refinement rather than outcome measurement, formal inter-rater agreement statistics were not calculated during scenario development.

The scenarios were designed to reflect realistic and commonly encountered clinical situations in daily implant practice rather than hypothetical or artificially simplified cases. Medical conditions in scenarios involving systemic diseases were explicitly defined. In the scenarios, ridge width and height were standardized using millimetric (mm) values; furthermore, distances to critical anatomical structures—such as the maxillary sinus, inferior alveolar nerve, and mental foramen—were clearly defined. This approach enabled the testing of the artificial intelligence’s capacity to provide defect-specific surgical recommendations (e.g., grafting, sinus augmentation) rather than merely general treatment plans.

All cases were formulated as open-ended clinical scenarios under the assumption that a real patient posed the questions. To ensure clarity and standardization, all scenarios were systematically reviewed by two authors for grammatical accuracy, content consistency, and clinical plausibility prior to submission to ChatGPT. Each scenario incorporated relevant clinical history, radiographic findings, and medical information typically considered during implant treatment planning. Given the exploratory nature of the study, formal pilot testing was not performed; however, expert review by experienced clinicians was used as a proxy to ensure realism and relevance.

Each scenario was presented individually to ChatGPT-5.2 in a new chat session using a standardized prompt: “Based on the following clinical scenario, what is the most appropriate implant-supported prosthetic treatment plan?” No additional guidance, follow-up prompts, corrections, or interactions were provided. All responses were generated independently, recorded verbatim, and stored separately for subsequent analysis. A representative clinical scenario and an example response generated by ChatGPT-5.2 are presented in Table 1.

**Table 1:** Example question and response from ChatGPT-5.2

**Scenario 1 (Patient with systemic disease):**	
The patient is a 68-year-old individual with a history of coronary artery disease, who underwent stent placement two years ago and is currently on dual antiplatelet therapy with clopidogrel and aspirin. Clinical and radiographic evaluation revealed edentulism in the regions of teeth 15, 16, and 17, with no evidence of bone deficiency that would contraindicate implant placement. Based on the following clinical scenario, what is the most appropriate implant-supported prosthetic treatment plan?	
**ChatGPT-5.2 response**	
The patient is a 68-year-old individual with a history of coronary artery disease who underwent stent placement two years prior and is currently maintained on dual antiplatelet therapy (clopidogrel and aspirin). Clinical and radiographic evaluation revealed partial edentulism in the right posterior maxillary region, involving teeth 15, 16, and 17, with no evidence of bone resorption or anatomical limitations that would contraindicate implant placement. Given the patient’s cardiovascular profile, perioperative bleeding risk must be carefully managed; thus, preoperative multidisciplinary coordination with the cardiology team is recommended to optimize antiplatelet therapy management. Surgically, placement of 2–3 dental implants in the posterior maxilla, corresponding to teeth 15–17, is proposed based on available bone volume and quality. Minimally invasive flap techniques and local hemostatic measures should be employed to minimize perioperative complications. An osseointegration period of 3–6 months is anticipated, during which regular clinical and radiographic follow-up is essential. Upon completion of osseointegration, restorative rehabilitation with an implant-supported fixed partial prosthesis is planned. To ensure long-term functional stability and biomechanical integrity, careful occlusal planning and selection of appropriate restorative materials, such as zirconia or porcelain-fused-to-metal, are warranted. Postoperative management should include regular professional maintenance, patient oral hygiene instruction, and annual radiographic assessment.	

### Evaluation of Reliability, Quality, and Safety

Given that the applied evaluation instruments rely on subjective expert judgment, inter-rater reliability was explicitly assessed to ensure methodological rigor and to minimize potential evaluator-related bias. All AI-generated responses were independently assessed by three board-certified oral and maxillofacial surgeons. The evaluation utilized the following scales (Table 2):

**Table 2:**
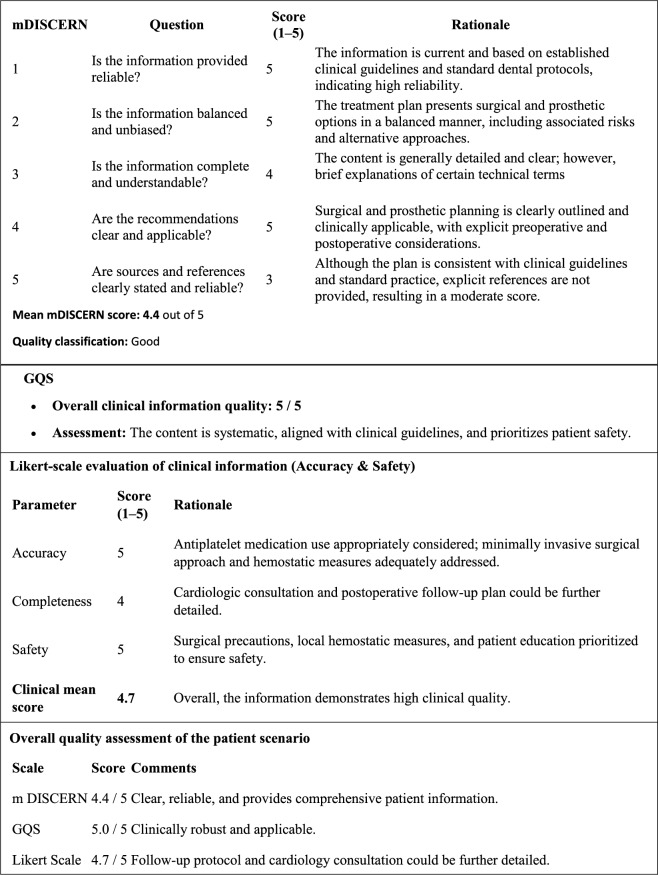
Summary of mDISCERN, GQS and Likert results of ChatGPT-5.2 responses after consensus score

- Modified DISCERN (mDISCERN) Scale: The reliability of information was evaluated using the first section of the DISCERN tool. Each item was scored as 1 (No), 2–4 (Partially), or 5 (Yes). The mean score across items for each scenario was calculated to determine the overall mDISCERN score, which was classified as poor (1.0–2.4), moderate (2.5–3.4), or good (3.5–5.0) [11].
- Global Quality Score (GQS): Overall quality was assessed on a 5-point scale, with 1 representing low quality and 5 representing high quality. The average score for each scenario was calculated and categorized as low (1–2), moderate (3), or high (4–5) [12].
- Likert Scale (Accuracy–Safety): Quality indicators such as accuracy and safety were measured using a 5-point Likert scale, where 1 indicated “completely incorrect/unsafe” and 5 indicated “completely correct/safe [13].”

Each response was evaluated in terms of clinical accuracy, patient safety, and adherence to standard implant protocols. A response was considered “completely correct and safe” (Likert score = 5) if the information provided was consistent with current evidence-based clinical guidelines, including ITI and EAO recommendations, contained no clinically misleading statements, and did not pose any potential risk to patient safety. Responses that included partially accurate information, omitted relevant clinical considerations, or contained potentially misleading or unsafe recommendations were scored accordingly at lower levels.

All responses were generated using the publicly available ChatGPT-5.2 model under default settings. To evaluate response reproducibility, each clinical scenario was submitted to ChatGPT-5.2 on three separate occasions, each conducted in a new chat session under identical conditions. No modifications were made to the prompt wording or input content across repetitions. Reproducibility was assessed by comparing the three responses generated for each scenario. Responses were classified as reproducible if the proposed treatment approach, implant-related recommendations, and safety considerations were consistent across all repetitions. Scenarios were classified as non-reproducible when clinically meaningful differences were observed between responses, including variations in treatment strategy, implant selection, need for additional surgical procedures, or safety-related information. All interactions were performed within a defined time period using the same publicly available model version to minimize potential variability related to system updates.

### Ethical approval and conduct of the study

This study was conducted in accordance with the ethical principles outlined in the Declaration of Helsinki. Only publicly available data generated by artificial intelligence were used, and no identifiable information from real patients or human participants was included. As the ChatGPT-5.2 model employed in this study is publicly accessible, formal institutional ethical approval was not required.

### Statistical analysis

This study was conducted on 60 scenarios. Data analysis was performed using IBM SPSS Statistics version 26.0. Frequency analyses were initially conducted to describe disease status. The normality of variables was assessed using the Shapiro–Wilk test. For variables that did not follow a normal distribution, the Mann–Whitney U test was applied. Relationships between non-normally distributed quantitative variables were evaluated using Spearman’s rank correlation coefficient.

All statistical tests were performed at a significance level of 5% (*p* < 0.05). Effect sizes for correlation analyses were reported as Spearman’s r together with 95% confidence intervals. As the analyses were exploratory and hypothesis-generating rather than confirmatory, no formal correction for multiple comparisons was applied. To evaluate the consistency and reliability of the scores assigned by the three independent board-certified surgeons, the Intraclass Correlation Coefficient (ICC) was employed. ICC values were interpreted according to established guidelines, where a value of 0.85 indicates strong inter-rater agreement. All exact p-values are reported to provide a transparent overview of the data.

## Results

A total of 60 clinical scenarios were analyzed, including 30 bone deficiency cases (50%) and 30 systemic disease cases (50%). Table 3 presents the comparison of mDISCERN GQS, Accuracy & Safety scores according to disease condition.

For bone deficiency scenarios, the mean mDISCERN score was 3.98 ± 0.21 (median: 4.00; range: 3.60–4.40). For systemic disease scenarios, the mean score was 4.13 ± 0.33 (median: 4.10; range: 3.60–4.80). No statistically significant difference was observed between groups (U = 560.50; *p* = 0.094).

The mean GQS score was 3.20 ± 0.40 (median: 3.00; range: 3.00–4.00) for bone deficiency scenarios and 3.83 ± 0.69 (median: 4.00; range: 3.00–5.00) for systemic disease scenarios. The difference between groups was statistically significant (U = 675.00; *p* < 0.001).

The mean Accuracy & Safety score was 4.18 ± 0.29 (median: 4.00; range: 3.70–4.70) in the bone deficiency group and 4.13 ± 0.43 (median: 4.33; range: 3.00–5.00) in the systemic disease group. No statistically significant difference was detected (U = 434.00; *p* = 0.805).

These findings indicate that mDISCERN and Accuracy & Safety scores were comparable across disease types, whereas GQS scores were significantly higher in systemic disease scenarios.

Table 4 presents the Spearman correlation coefficients among mDISCERN, GQS, and Accuracy & Safety scores for all observations. A weak but statistically significant positive correlation was observed between mDISCERN and GQS (*r* = 0.325; *p* = 0.011). A moderate positive correlation was found between mDISCERN and Accuracy & Safety (*r* = 0.535; *p* < 0.001). No significant correlation was identified between GQS and Accuracy & Safety (*r* = 0.056; *p* = 0.673).

Table 5 presents the correlation analysis among the evaluation scales within the bone deficiency subgroup. In the bone deficiency group, moderate to strong positive correlations were observed across all scales. mDISCERN was moderately correlated with GQS (*r* = 0.512; *p* = 0.004) and strongly correlated with Accuracy & Safety (*r* = 0.651; *p* < 0.001). GQS and Accuracy & Safety also demonstrated a strong positive correlation (*r* = 0.682; *p* < 0.001).

Table 6 presents the correlation analysis among the evaluation scales within the systemic disease subgroup. In the systemic disease group, mDISCERN was not significantly correlated with GQS (*r* = 0.118; *p* = 0.535). A moderate positive correlation was observed between mDISCERN and Accuracy & Safety (*r* = 0.473; *p* = 0.008). No statistically significant correlation was found between GQS and Accuracy & Safety (*r* = − 0.288; *p* = 0.123).

**Table 3:** Comparison of mDISCERN GQS, Accuracy & Safety scores according to disease condition

Parameter	Disease status	Mean + SD	Median	Min - Max	U	*P*-value
mDISCERN	Bone deficiency	3.98 + 0.21	4.00	3.60–4.40	560.50	*P* = 0.094
	Systemic disease	4.13 + 0.33	4.10	3.60–4.80		
GQS	Bone deficiency	3.20 + 0.40	3.00	3.00–4.00	675.00	*P* < 0.001*
	Systemic disease	3.83 + 0.69	4.00	3.00–5.00		
Accuracy and safety	Bone deficiency	4.18 + 0.29	4.00	3.70–4.70	434.00	*P* = 0.805
	Systemic disease	4.13 + 0.43	4.33	3.00–5.00		

*Min* Minimum, *Max* Maximum, *SD* Standart deviation, *mDISCERN* Modified DISCERN GQS Global Quality Scale Mann Whitney U Test, * *p*<0.05

**Table 4:** Correlation results among scales (All observations)

		m DISCERN	GQS	Accuracy & Safety
Modified DISCERN	r	1.00	0.325*	0.535*
	p		0.011	0.000
GQS	r		1.00	0.056
	p			0.673
Accuracy & Safety	r			1.00
	p			

*mDISCERN* Modified DISCERN, *GQS* Global Quality Scale Correlation coefficients are presented as r values with 95% confidence intervals (CI); *p* < 0.05 was considered statistically significant

r: Spearman’s rho correlation analysis

**Table 5:** Correlation results among scales (Participants in the bone insufficiency scenario group)

		mDISCERN	GQS	Accuracy & Safety
mDISCERN	r	1.00	0.512*	0.651*
	p		0.004	0.000
GQS	r		1.00	0.682*
	p			0.000
Accuracy & Safety	r			1.00

*mDISCERN* Modified DISCERN, *GQS* Global Quality Scale. Correlation coefficients are presented as r values with 95% confidence intervals (CI); *p* < 0.05 was considered statistically significant

r: Spearman’s rho correlation analysis

**Table 6:** Correlation results among scales (Participants with systemic disease scenario group)

		mDISCERN	GQS	Accuracy & Safety
mDISCERN	r	1.00	0.118	0.473*
	p		0.535	0.008
GQS	r		1.00	−0.288
	p			0.123
Accuracy & Safety	r			1.00

*mDISCERN* Modified DISCERN, *GQS* Global Quality Scale. Correlation coefficients are presented as r values with 95% confidence intervals (CI); *p* < 0.05 was considered statistically significant﻿

r: Spearman’s rho correlation analysis

## Di̇scussi̇on

Despite the increasing use of LLMs for generating clinical information, there are notable limitations regarding their reliability and accuracy. In dentistry, ChatGPT and comparable models often provide responses with high confidence; however, they can produce inaccurate or misleading information, commonly referred to as “hallucinations.” Indeed, previous studies have reported that 5–13% of LLM-generated responses to medical questions may be potentially harmful or unreliable [14]. Moreover, the occasional inability of these models to provide verifiable or literature-based references is considered a significant risk factor, particularly in clinical decision-making processes [15]. In this study, responses generated by ChatGPT-5.2 to fictional patient scenarios were systematically analyzed using the mDISCERN, GQS, and accuracy/safety parameters. The findings indicate that the model generally provides safe, consistent, and clinically verifiable information, particularly in scenarios involving bone insufficiency and systemic diseases. Evaluations based on the mDISCERN, GQS, and accuracy/safety scales revealed that the majority of responses met moderate to high standards of reliability and quality. Furthermore, the high correlation observed among responses obtained across different sessions suggests that the model demonstrates a strong capacity for reproducibility. This result is consistent with previous studies in the literature suggesting that LLMs may function as supportive tools for patient education and general health information dissemination. For instance, a prospective study reported that chatbot-generated responses achieved high levels of accuracy and completeness when compared with physician-provided answers to medical inquiries [16]. Such findings highlight the potential of LLMs to enhance health literacy, facilitate access to information, and support dentist–patient communication. In addition, some users and healthcare professionals have reported that, based on LLM-generated responses, ChatGPT is useful for rapid access to medical information, general knowledge acquisition, and preliminary patient briefing purposes [17]. This highlights the potential advantages of LLMs, particularly for individuals with limited access to healthcare resources or lower levels of health literacy. When the findings of the present study are considered alongside supportive evidence from the literature, it appears that ChatGPT-5.2 and similar LLMs may be cautiously and supportively utilized as tools for patient education, information provision, and preliminary assessment.

The strong positive correlations observed among all three evaluation scales in bone insufficiency scenarios reflect ChatGPT-5.2’s capability to generate accurate and reliable information in standardized clinical contexts. These findings are consistent with previous studies suggesting that large language models can be effectively integrated into patient education and informational processes in routine clinical scenarios [16, 18]. Similarly, several studies have reported that ChatGPT demonstrates satisfactory overall quality and safety performance in scenarios related to oral surgery and dental implantology [19, 20].

In conclusion, although the responses generated by ChatGPT in this study were generally found to be safe and accurate, existing literature indicates that model reliability may decrease in complex and highly variable clinical scenarios [14, 15].

Conversely, in the present study, the attenuation of inter-scale correlations observed in more complex scenarios involving systemic diseases suggests that the model may exhibit limitations in terms of accuracy and reliability when addressing complex clinical conditions. The literature indicates that LLMs may demonstrate reduced response quality in rare or highly variable clinical situations and carry a risk of generating incorrect or fabricated information, commonly referred to as “hallucinations” [21, 22]. This underscores the necessity of positioning AI-based models not as autonomous authorities in clinical decision-making, but rather as supportive and informational tools under professional supervision.

Consistent with these observations, the systemic disease scenario group in our study demonstrated lower inter-scale correlation coefficients compared with the bone insufficiency group. This finding aligns with existing evidence and further suggests that LLM performance may be constrained as clinical complexity increases.

Another critical concern associated with LLMs is the tendency for users to perceive their responses as highly trustworthy. The literature indicates that users may attribute excessive confidence to LLM-generated information, even when accuracy is limited, thereby increasing the risk of misinformation. Moreover, several studies have demonstrated that such responses can influence patient decision-making and may even lead individuals to pursue treatment without seeking professional consultation [23].

The findings of the present study are consistent with reports in the literature highlighting the potential of LLMs to provide reliable, coherent, and comprehensible information [14, 23]. However, despite the overall consistency and accuracy of ChatGPT responses observed in this study, the possibility that the model’s appearance of reliability may exert a misleading effect on user perception should not be overlooked. Furthermore, our findings indicating reduced performance in complex clinical scenarios align with contrasting evidence reported in the literature [24, 25].

In addition, several critical warnings regarding the medical use of large language models have been emphasized in previous research. Studies have shown that responses generated by popular chatbots to patient queries may contain “potentially unsafe or harmful” content in approximately 5–13% of cases [14]. Similarly, a comprehensive meta-analysis reported that the average accuracy of LLM responses to medical queries remains at approximately 56% [21]. Collectively, these findings suggest that the clinical accuracy of current LLMs has not yet reached a threshold that would justify their use as fully reliable standalone tools.

In addition, users have been shown to exhibit a tendency to place high levels of trust in responses with relatively low accuracy rates [23]. This perception of overconfidence increases the risk of misinformation and may contribute to potential clinical harm. The literature further emphasizes that these models are capable of computational errors, may suggest inappropriate treatment protocols, and, most critically, may generate non-existent or fabricated literature references commonly referred to as “hallucinations” [17].

These findings indicate that while LLMs hold considerable potential as supportive, informative, and reproducible tools in healthcare and dental settings, their use as standalone instruments in clinical decision-making remains risky. Accordingly, the interpretation of model-generated outputs should always be conducted under the supervision of qualified healthcare professionals.

The satisfactory overall reliability and quality performance of LLM responses in the context of dental implantology and bone insufficiency is encouraging. However, dental practice, particularly implant treatment planning, constitutes a highly sensitive and complex process that requires the simultaneous evaluation of numerous variables, including bone quality and volume, periodontal status, systemic conditions, tobacco and alcohol use, patient age, and detailed medical history. This multifactorial clinical framework may render the limitations of LLM-based responses more pronounced with respect to diagnostic accuracy and patient safety. Indeed, the literature reports that, in response to patient questions related to dental and maxillofacial surgery, ChatGPT-4 demonstrates sufficient readability but exhibits limited performance in terms of empathy, clinical appropriateness, and safety [26]. Similarly, studies indicate that healthcare-specific chatbots provide higher reliability and readability scores compared with ChatGPT [27]. In the absence of fundamental clinical information, such as physical examination findings and laboratory or radiographic data, the ability of LLMs to perform accurate clinical assessment and decision-making is naturally constrained [21]. The current literature emphasizes that artificial intelligence based systems, such as.

LLMs, should be approached with caution in clinical applications. Numerous studies highlight that LLMs may not fully comprehend the specific clinical context, can generate inconsistent or erroneous information, and may lead to critical errors when integrated into actual clinical decision-making, indicating that these technologies are not inherently reliable for direct clinical decisions. For example, a systematic review reported that LLMs demonstrate limitations in key areas such as reliability, consistency, and adherence to clinical guidelines, underscoring the need for careful evaluation [24]. Moreover, retrospective studies report that LLMs struggle to comply with established clinical guidelines in tasks such as treatment planning, and therefore are not suitable for independently guiding therapeutic decisions [28]. These findings indicate that erroneous recommendations in critical clinical domains, such as drug dosing, surgical indications, or chronic disease management, may pose serious risks. Despite demonstrating good diagnostic accuracy, the authors concluded that ChatGPT may serve as a useful adjunct for clinicians in complex cases, but should be used cautiously by non-professionals. In other words, it holds potential as a clinical decision support tool rather than as a standalone diagnostic instrument [29, 30] Current evidence indicates that in procedures involving numerous variables, such as dental implants, LLMs are often unable to adequately assess patient-specific factors and multimodal data, tending instead to provide generalized information. This limitation becomes particularly relevant in complex clinical scenarios that require individualized treatment planning [25]. Although accurate information was generated in the dental implant and bone insufficiency scenarios in the present study, the literature clearly demonstrates that LLMs may produce incomplete or erroneous information in more complex and personalized clinical contexts.

The findings of the present study should be interpreted within the broader international literature evaluating large language models in dental education and clinical contexts. Recent studies have increasingly focused on the performance of generative AI systems across different dental disciplines, including licensure examinations, restorative dentistry, pediatric dentistry, and oral oncology.

Chau et al. evaluated the performance of generative AI models in dentistry licensure examinations and demonstrated that, while LLMs can achieve moderate to high accuracy in knowledge-based multiple-choice questions, their performance varies considerably depending on question complexity and clinical nuance [31]. Similar results were reported in subsequent studies assessing chatbot responses in prosthodontics and restorative dentistry, where LLMs showed acceptable factual accuracy but limited reasoning consistency in clinically demanding scenarios [32]. These findings align with the present study, in which reproducibility was observed under standardized conditions, but performance declined as clinical complexity increased.

In pediatric dentistry, Rokhshad et al. reported that chatbot-generated responses demonstrated lower accuracy and consistency compared with clinician-provided answers, particularly in patient-specific and behavior-sensitive scenarios [33]. Furthermore, systematic evaluations of chatbots for conducting pediatric dentistry reviews highlighted limitations in methodological rigor, contextual understanding, and clinical judgment [34]. These observations are consistent with our subgroup findings, suggesting that LLM reliability diminishes in heterogeneous and complex clinical contexts, such as those involving systemic diseases.

Studies evaluating AI chatbots in oral oncology further emphasize this limitation. Although chatbots were found to provide coherent and empathetic responses to frequently asked questions, their clinical accuracy and depth of contextual understanding remained variable [35]. Collectively, these international findings support the conclusion that LLMs perform best in structured, knowledge-based tasks, while their reliability decreases in scenarios requiring individualized clinical reasoning and integration of multifactorial patient data.

Taken together, the present study is consistent with the growing body of international evidence suggesting that LLMs should be regarded as supportive informational tools rather than independent clinical decision-making systems. While their reproducibility and accessibility may offer value for patient education and preliminary information provision, professional oversight remains essential to ensure patient safety and clinical appropriateness.

The findings of the present study should be interpreted within the broader international literature evaluating large language models (LLMs) and artificial intelligence–based systems in dentistry and radiographic analysis. In recent years, an increasing number of studies have examined the performance of generative AI systems across different dental and medical imaging disciplines.

In a study evaluating the diagnostic performance of ChatGPT-4o in knee osteoarthritis radiographs, the model demonstrated high sensitivity in positive–negative discrimination but showed limited accuracy in detailed grading tasks [36]. Similarly, a comparative study assessing AI-based ChatGPT models in the analysis of hand–wrist radiographs reported acceptable overall accuracy; however, the depth of clinical interpretation and detailed analytical capability remained inferior to expert evaluation [37]. These findings parallel the results of the present study, where correlation coefficients decreased in more complex scenarios involving systemic diseases.

In contrast, the CBMNet study, which evaluated a dual-attention enhanced ConvNeXt model for GV Black type I–III classification in intraoral periapical radiographs, reported high classification accuracy in structured imaging tasks [38]. This suggests that task-specific, image-trained deep learning models may achieve more consistent performance in narrowly defined classification problems compared with general-purpose LLMs.

Furthermore, in a study assessing AI-driven large language models for orthodontic aesthetic scoring using the IOTN-AC index, the models demonstrated acceptable scoring performance but exhibited limitations in clinical standardization and detailed grading precision [39]. These observations are consistent with the present findings, which indicate moderate consistency under standardized conditions but reduced reliability as clinical complexity increases.

Collectively, the international evidence indicates that both LLM-based systems and image-processing AI models can perform satisfactorily in structured and well-defined tasks. However, their reliability decreases in multifactorial clinical decision-making contexts requiring individualized reasoning and integration of complex patient data. This broader literature supports the conclusion of the present study that LLMs should be positioned as supportive informational tools rather than independent clinical decision-making systems.

Current evidence indicates that the performance of LLMs is relatively higher for questions concerning adult and general populations, whereas it decreases for pediatric patients, older adults, or individuals with severe systemic conditions, highlighting the lack of comprehensive age-specific clinical data in model training [40]. Furthermore, the static nature of the training datasets, which may not consistently include up-to-date clinical guidelines or local treatment protocols, represents a significant limitation, as LLMs may not adapt to evolving best practices over time [16].

The tendency of users to develop “over-reliance” on LLM responses, along with the models’ limited ability to substantiate information with scientific references, has been identified as a critical limitation requiring careful consideration during use [28, 40]. Therefore, it is essential that model outputs are verified by a qualified clinician prior to clinical application. Although the use of standardized and hypothetical scenarios in the present study enhanced the consistency of results, it should be acknowledged that the heterogeneity of real patient populations may negatively impact LLM performance. The data obtained from our study align with current literature supporting the potential of LLMs to provide reliable, consistent, and comprehensible information in healthcare and dentistry [14, 23]Performance fluctuations observed in complex scenarios corroborate the cautious approach highlighted in previous studies [24, 25]. In conclusion, ChatGPT-4 and similar models demonstrate substantial potential as adjunctive tools with high reproducibility for dental education and patient information purposes; however, their use in direct clinical decision-making should remain limited due to the inherent risks involved. Our choice of 60 clinical scenarios aligns with recent studies in the field, such as the work by Chau et al. (2024), which emphasized the importance of using structured, expert-validated cases rather than raw volume to assess AI’s clinical reasoning capabilities [31]. This sample size allowed for a detailed, triple-blind expert review process, ensuring that each response was meticulously analyzed for both accuracy and patient safety.

One limitation of our study is the relatively focused sample size of 60 scenarios. Although this number provided a wide range of clinical variables, future studies with larger, multi-center datasets or more granular scenarios could further enhance the generalizability of these findings. Finally, our analysis did not include adjustments for multiple comparisons. While this approach is consistent with the exploratory objective of characterizing AI performance across diverse metrics, the results should be interpreted with an awareness of the potential for Type I error inflation.”

## Conclusion

This study provides an exploratory evaluation of ChatGPT-5.2’s performance in dental implant treatment planning. The findings suggest that the model holds the potential to provide generally reliable, consistent, and comprehensible information in structured clinical scenarios, such as bone deficiencies. However, these results should be interpreted within the specific context of the 60 clinical scenarios evaluated and the current model version, avoiding overgeneralization. The analysis revealed that as clinical complexity increases—particularly in cases involving multifactorial systemic conditions—the correlation between evaluation scales tends to weaken. This finding suggests that despite their ability to synthesize theoretical knowledge, large language models (LLMs) may not yet offer expert-level precision in situations requiring individualized risk assessment and complex clinical reasoning.

In conclusion, while ChatGPT can be positioned as a promising adjunctive tool for patient education and rapid information retrieval in dental practice, it should not be regarded as a standalone authority to replace clinical decision-making mechanisms. The model’s role can be likened to that of an “exceptionally intelligent assistant who has read millions of books but has never seen a patient.” Therefore, model outputs must always be verified under the supervision of an experienced clinician, in light of patient-specific clinical parameters and current evidence-based guidelines.

## Supplementary Information

Supplementary Material 1.

## Data Availability

The data that support the findings of this study are available from the corresponding author upon reasonable request.

## Abbreviations

AI: Artificial Intelligence
LLMs: Large language models
ChatGPT: Chat Generative Pretrained Transformer
GQS: Global Quality scale
mDISCERN: Modified DISCERN
ITI: International Team for Implantology
EAO: European Association for Osseointegration
SPSS: Statistical package for the social sciences
IOTN-AC: Index of Orthodontic Treatment Need – Aesthetic Component
CBMNet: Convolutional Block-based Multi-attention Network
GV: Greene Vardiman

## References

[1] Topol EJ. High-performance medicine: the convergence of human and artificial intelligence. Nat Med. 2019;25:44–56. 10.1038/s41591-018-0300-7. 30617339 10.1038/s41591-018-0300-7

[2] Esteva A, Robicquet A, Ramsundar B, Kuleshov V, DePristo M, Chou K, et al. A guide to deep learning in healthcare. Nat Med. 2019;25:24–9. 10.1038/s41591-018-0316-z. 30617335 10.1038/s41591-018-0316-z

[3] Jiang F, Jiang Y, Zhi H, Dong Y, Li H, Ma S, et al. Artificial intelligence in healthcare: past, present and future. Stroke Vasc Neurol. 2017;2:230–43. 10.1136/svn-2017-000101. 29507784 10.1136/svn-2017-000101 PMC5829945

[4] Şişman AÇ, Acar AH. Artificial intelligence-based chatbot assistance in clinical decision-making for medically complex patients in oral surgery: a comparative study. BMC Oral Health. 2025;25:351. 10.1186/s12903-025-05732-w. 40055745 10.1186/s12903-025-05732-w PMC11887094

[5] Siden R, Kerman H, Gallo RJ, Cool JA, Hom J, Goh E, et al. A typology of physician input approaches to using AI chatbots for clinical decision-making. Npj Digit Med. 2025. 10.1038/s41746-025-02184-y. 41350807 10.1038/s41746-025-02184-y PMC12780094

[6] Srikanth G, Singh A, Kudva A. Treatment planning for single-tooth implant: a clinical guide and literature review. J Maxillofac Oral Surg. 2025;24:886–98. 10.1007/s12663-025-02631-z. 40756933 10.1007/s12663-025-02631-z PMC12316644

[7] Arbildo-Vega HI, Cruzado-Oliva FH, Infantes-Ruíz ED, Luján-Valencia SA, Meza-Málaga JM, Marroquín-Soto C, et al. Clinical performance of short implants vs. standard implants in edentulous patients. An umbrella review. Front Oral Health. 2025;6:1670095. 10.3389/froh.2025.1670095. 41050840 10.3389/froh.2025.1670095 PMC12488567

[8] Zhu S, Chen R, Zhang S, Wang Z, Xie Z. Outcomes of dental implant treatment in 3–18 year old children with ectodermal dysplasia: a systematic review spanning three decades. BMC Oral Health. 2025;25:1818. 10.1186/s12903-025-07230-5. 41272663 10.1186/s12903-025-07230-5 PMC12639673

[9] Hu H, Liu L, Man Y, Ye Z, You M. Clinical and radiologic outcomes of dental implants in cemento-osseous dysplasia: a systematic review and retrospective case series. BMC Oral Health. 2025;25:1117. 10.1186/s12903-025-06509-x. 40618148 10.1186/s12903-025-06509-x PMC12228152

[10] Elagib MFA, Alqaysi MAH, Almushayt MOS, Nagate RR, Gokhale ST, Chaturvedi S. Dental implants in growing patients: a systematic review and meta-analysis. Technol Health Care Off J Eur Soc Eng Med. 2023;31:1051–64. 10.3233/THC-220581. 10.3233/THC-220581 36502352

[11] Charnock D, Shepperd S, Needham G, Gann R. DISCERN: an instrument for judging the quality of written consumer health information on treatment choices. J Epidemiol Community Health. 1999;53:105–11. 10.1136/jech.53.2.105. 10396471 10.1136/jech.53.2.105 PMC1756830

[12] Bernard A, Langille M, Hughes S, Rose C, Leddin D, van Veldhuyzen Zanten S. A systematic review of patient inflammatory bowel disease information resources on the World Wide Web. Am J Gastroenterol. 2007;102:2070–7. 10.1111/j.1572-0241.2007.01325.x. 17511753 10.1111/j.1572-0241.2007.01325.x

[13] Likert R. A technique for the measurement of attitudes. Arch Psychol. 1932;22:140:55–55.

[14] Draelos RL, Afreen S, Blasko B, Brazile TL, Chase N, Desai DP, et al. Large language models provide unsafe answers to patient-posed medical questions. 2025. 10.48550/arXiv.2507.18905. 10.1038/s41746-026-02428-5 PMC13013898 41688533

[15] Shiferaw MW, Zheng T, Winter A, Mike LA, Chan L-N. Assessing the accuracy and quality of artificial intelligence (AI) chatbot-generated responses in making patient-specific drug-therapy and healthcare-related decisions. BMC Med Inform Decis Mak. 2024;24:404. 10.1186/s12911-024-02824-5. 39719573 10.1186/s12911-024-02824-5 PMC11668057

[16] Goodman RS, Patrinely JR, Stone CA, Zimmerman E, Donald RR, Chang SS, et al. Accuracy and reliability of chatbot responses to physician questions. JAMA Netw Open. 2023;6:e2336483–2336483. 37782499 10.1001/jamanetworkopen.2023.36483 PMC10546234

[17] Shiferaw MW, Zheng T, Winter A, Mike LA, Chan L-N. Assessing the accuracy and quality of artificial intelligence (AI) chatbot-generated responses in making patient-specific drug-therapy and healthcare-related decisions. BMC Med Inform Decis Mak. 2024;24:404. 10.1186/s12911-024-02824-5. 39719573 10.1186/s12911-024-02824-5 PMC11668057

[18] Chen S-Y, Kuo HY, Chang S-H. Perceptions of ChatGPT in healthcare: usefulness, trust, and risk. Front Public Health. 2024;12:1457131. 39346584 10.3389/fpubh.2024.1457131 PMC11436320

[19] Alsayed AA, Aldajani MB, Aljohani MH, Alamri H, Alwadi MA, Alshammari BZ, et al. Assessing the quality of AI information from ChatGPT regarding oral surgery, preventive dentistry, and oral cancer: an exploration study. Saudi Dent J. 2024;36:1483–9. 39619711 10.1016/j.sdentj.2024.09.009 PMC11605724

[20] Tuzlalı M, Baki N, Aral K, Aral CA, Bahçe E. Evaluating the performance of AI chatbots in responding to dental implant FAQs: a comparative study. BMC Oral Health. 2025;25:1548. 10.1186/s12903-025-06863-w. 41063105 10.1186/s12903-025-06863-w PMC12505636

[21] Wei Q, Yao Z, Cui Y, Wei B, Jin Z, Xu X. Evaluation of ChatGPT-generated medical responses: a systematic review and meta-analysis. J Biomed Inform. 2024;151:104620. 38462064 10.1016/j.jbi.2024.104620

[22] Hao W-R, Chen C-C, Chen K, Li L-C, Chiu C-C, Yang T-Y, et al. ChatGPT performance deteriorated in patients with comorbidities when providing cardiological therapeutic consultations. Healthcare. 2025. 10.3390/healthcare13131598. 10.3390/healthcare13131598 PMC12249446 40648622

[23] Shekar S, Pataranutaporn P, Sarabu C, Cecchi GA, Maes P. People over trust AI-generated medical responses and view them to be as valid as doctors, despite low accuracy. 2024. 10.48550/arXiv.2408.15266.

[24] Shool S, Adimi S, Saboori Amleshi R, Bitaraf E, Golpira R, Tara M. A systematic review of large language model (LLM) evaluations in clinical medicine. BMC Med Inform Decis Mak. 2025;25:117. 10.1186/s12911-025-02954-4. 40055694 10.1186/s12911-025-02954-4 PMC11889796

[25] Wu X, Cai G, Guo B, Ma L, Shao S, Yu J, et al. A multi-dimensional performance evaluation of large language models in dental implantology: comparison of ChatGPT, DeepSeek, Grok, Gemini and Qwen across diverse clinical scenarios. BMC Oral Health. 2025;25:1272. 10.1186/s12903-025-06619-6. 40721763 10.1186/s12903-025-06619-6 PMC12302792

[26] Othman AA, Sharqawi AJ, MohammedAziz AA, Ali WA, Alatiyyah AA, Mirah MA, et al. Assessing the accuracy and completeness of AI-generated dental responses: an evaluation of the Chat-GPT model. Healthcare. 2025. 10.3390/healthcare13172144. 40941502 10.3390/healthcare13172144 PMC12428179

[27] Singhal K, Azizi S, Tu T, Mahdavi SS, Wei J, Chung HW, et al. Large language models encode clinical knowledge. Nature. 2023;620:172–80. 37438534 10.1038/s41586-023-06291-2 PMC10396962

[28] Roeschl T, Hoffmann M, Hashemi D, Rarreck F, Hinrichs N, Trippel TD, et al. Assessing the Limitations of Large Language Models in Clinical Practice Guideline–Concordant Treatment Decision-Making on Real-World Data: Retrospective Study. JMIRx Med. 2025;6:e74899. 10.2196/74899. 41190890 10.2196/74899 PMC12587749

[29] Mehnen L, Gruarin S, Vasileva M, Knapp B. ChatGPT as a medical doctor? A diagnostic accuracy study on common and rare diseases. MedRxiv. 2023;2023–04. 10.1101/2023.04.20.23288859.

[30] Isleyen M, Aydemir ME. How useful are YouTube videos on labial frenectomy? A quality review. BMC Oral Health. 2025;25:1434. 10.1186/s12903-025-06786-6. 41013428 10.1186/s12903-025-06786-6 PMC12465663

[31] Chau RCW, Thu KM, Yu OY, Hsung RTC, Lo ECM, Lam WYH. Performance of generative artificial intelligence in dental licensing examinations. Int Dent J. 2024;74(3):616–21. 10.1016/j.identj.2023.11.002. 38242810 10.1016/j.identj.2023.12.007 PMC11123518

[32] Chau RCW, Thu KM, Yu OY, Hsung RTC, Wang DCP, Man MWH, et al. Evaluation of Chatbot Responses to Text-Based Multiple-Choice Questions in Prosthodontic and Restorative Dentistry. Dent J. 2025;13(1):12. 10.3390/dj13010012. 10.3390/dj13070279 PMC12293279 40710124

[33] Rokhshad R, Zhang P, Mohammad-Rahimi H, Pitchika V, Entezari N, Schwendicke F. Accuracy and consistency of chatbots versus clinicians for answering pediatric dentistry questions: a pilot study. J Dent. 2024;144:104938. 10.1016/j.jdent.2024.104938. 38499280 10.1016/j.jdent.2024.104938

[34] Rokhshad R, Mohammad FD, Nomani M, Mohammad-Rahimi H, Schwendicke F. Chatbots for conducting systematic reviews in pediatric dentistry. J Dent. 2025;158:105733. 10.1016/j.jdent.2025.105733. 40194755 10.1016/j.jdent.2025.105733

[35] Rokhshad R, Khoury ZH, Mohammad-Rahimi H, Motie P, Price JB, Tavares T, et al. Efficacy and empathy of AI chatbots in answering frequently asked questions on oral oncology. Oral Surg Oral Med Oral Pathol Oral Radiol. 2025;139(6):719–28. 10.1016/j.oooo.2024.11.012. 39843286 10.1016/j.oooo.2024.12.028

[36] Zhu J, Jiang Y, Chen D, Lu Y, Huang Y, Lin Y, et al. High identification and positive-negative discrimination but limited detailed grading accuracy of ChatGPT-4o in knee osteoarthritis radiographs. Knee Surg Sports Traumatol Arthrosc. 2025;33:1911–9. 10.1002/ksa.12639. 40053915 10.1002/ksa.12639

[37] Yıldırım A, Cicek O, Genç YS. Can AI-based ChatGPT models accurately analyze hand–wrist radiographs? A comparative study. Diagnostics. 2025;15(12):1513. 10.3390/diagnostics15121513. 40564836 10.3390/diagnostics15121513 PMC12191842

[38] Parathanath A, Manimaran A. CBMNet: a dual-attention enhanced ConvNeXt model for accurate G.V. Black type I-III classification in intraoral periapical radiographs. Sci Rep. 2025;15:39287. 10.1038/s41598-025-39287-x. 41214050 10.1038/s41598-025-22975-3 PMC12603191

[39] Yıldırım A, Cicek O. Assessment of AI-driven large language models for orthodontic aesthetic scoring using the IOTN-AC. Diagnostics. 2025;15(23):3048. 10.3390/diagnostics15233048. 41374429 10.3390/diagnostics15233048 PMC12691508

[40] Mondillo G, Perrotta A, Frattolillo V, Colosimo S, Indolfi C, del Giudice MM, et al. Large language models performance on pediatrics question: a new challenge. J Med Artif Intell. 2025;8. 10.21037/jmai-24-174.

