# Supplementary material for: Performance of ChatGPT in dental implant treatment planning: evaluation using the modified DISCERN, Global Quality Score, and accuracy–safety score
Source: BMC Oral Health. 2026 Mar 28;26:966. doi: 10.1186/s12903-026-08009-y (PMC13242144; doi:10.1186/s12903-026-08009-y)
Supplement: Supplementary file 1 — Supplementary Material 1. [file 12903_2026_8009_MOESM1_ESM.docx]

**Part I: Systemic Condition Scenarios**

1. Patient Case: A 48-year-old patient presents with a systemic history of hypertension. Clinical examination reveals partial edentulism in the 35–37 region. Based on the following clinical scenario, what is the most appropriate implant-supported prosthetic treatment plan?
2. Patient Case: A 50-year-old patient has a medical history significant for infective endocarditis 3 years ago. The patient presents with a single tooth agenesis at position 16. Based on the following clinical scenario, what is the most appropriate implant-supported prosthetic treatment plan?
3. Patient Case: A 60-year-old patient underwent heart valve replacement 3 years ago and is currently receiving warfarin sodium therapy. Only teeth 13, 12, and 27 are present in the maxilla; the mandible is completely edentulous. Based on the following clinical scenario, what is the most appropriate implant-supported prosthetic treatment plan?
4. Patient Case: A 50-year-old patient is diagnosed with Type 2 Diabetes Mellitus with a recorded HbA1c of 7.6%. Edentulism is localized to the 21, 22, and 23 regions of the maxilla. Based on the following clinical scenario, what is the most appropriate implant-supported prosthetic treatment plan?
5. Patient Case: A 55-year-old patient diagnosed with rheumatoid arthritis is currently undergoing corticosteroid therapy. Edentulous spans are present at 24, 25, and 26 in the maxilla and 36–37 in the mandible. Based on the following clinical scenario, what is the most appropriate implant-supported prosthetic treatment plan?
6. Patient Case: A 67-year-old patient with osteoporosis has been using denosumab actively for 3 years. The maxillary arch is partially edentulous (16, 17, 26 missing), and the mandibular arch is completely edentulous. Based on the following clinical scenario, what is the most appropriate implant-supported prosthetic treatment plan?
7. Patient Case: A 48-year-old patient with a history of oral squamous cell carcinoma received chemotherapy and radiotherapy 2 years ago. Clinical findings show tooth loss in the right mandibular region. Based on the following clinical scenario, what is the most appropriate implant-supported prosthetic treatment plan?
8. Patient Case: A 42-year-old patient is diagnosed with anemia. Tooth 22 is missing in the maxilla. Based on the following clinical scenario, what is the most appropriate implant-supported prosthetic treatment plan?
9. Patient Case: A 58-year-old patient is diagnosed with epilepsy. Teeth 24, 25, 26, and 27 are missing. Based on the following clinical scenario, what is the most appropriate implant-supported prosthetic treatment plan?
10. Patient Case: A 39-year-old patient is receiving treatment for hypothyroidism. Teeth 35, 36, and 37 are missing. Based on the following clinical scenario, what is the most appropriate implant-supported prosthetic treatment plan?
11. Patient Case: A 77-year-old patient with osteoporosis has used oral alendronate for 3 years. Teeth 36 and 37 exhibit Grade 3 mobility, while teeth 45, 46, and 47 are missing. Based on the following clinical scenario, what is the most appropriate implant-supported prosthetic treatment plan?
12. Patient Case: A 56-year-old patient is a Hepatitis B carrier. Teeth 12, 13, and 14 are missing. Based on the following clinical scenario, what is the most appropriate implant-supported prosthetic treatment plan?
13. Patient Case: A 46-year-old patient diagnosed with Systemic Lupus Erythematosus (SLE) is currently on steroid therapy. Teeth 44, 45, and 46 are missing. Based on the following clinical scenario, what is the most appropriate implant-supported prosthetic treatment plan?
14. Patient Case: A 59-year-old patient has a history of non-head and neck radiotherapy. There is a single tooth deficiency at position 36. Based on the following clinical scenario, what is the most appropriate implant-supported prosthetic treatment plan?
15. Patient Case: A 55-year-old patient with rheumatoid arthritis is on corticosteroid treatment. Teeth 24, 25, and 26 are missing in the maxilla. Based on the following clinical scenario, what is the most appropriate implant-supported prosthetic treatment plan?
16. Patient Case: A 69-year-old patient with a history of atrial fibrillation and pulmonary embolism. The patient uses dabigatran and aspirin; teeth 46 and 47 are missing. Based on the following clinical scenario, what is the most appropriate implant-supported prosthetic treatment plan?
17. Patient Case: A 44-year-old patient with renal failure is undergoing dialysis. Teeth 16 and 17 are missing. Based on the following clinical scenario, what is the most appropriate implant-supported prosthetic treatment plan?
18. Patient Case: A 70-year-old patient is diagnosed with liver cirrhosis. The mandible is completely edentulous. Based on the following clinical scenario, what is the most appropriate implant-supported prosthetic treatment plan?
19. Patient Case: A 57-year-old patient is diagnosed with Chronic Obstructive Pulmonary Disease (COPD). The mandible is completely edentulous. Based on the following clinical scenario, what is the most appropriate implant-supported prosthetic treatment plan?
20. Patient Case: A 68-year-old patient with coronary artery disease had a stent placed 2 years ago and currently uses clopidogrel and aspirin. Teeth 15, 16, and 17 are missing. Based on the following clinical scenario, what is the most appropriate implant-supported prosthetic treatment plan?
21. Patient Case: A 62-year-old patient underwent heart valve prosthesis surgery 5 months ago and regularly uses warfarin. The mandible is completely edentulous. Based on the following clinical scenario, what is the most appropriate implant-supported prosthetic treatment plan?
22. Patient Case: A 51-year-old patient presents with systemic arrhythmia. Teeth 46 and 47 are missing. Based on the following clinical scenario, what is the most appropriate implant-supported prosthetic treatment plan?
23. Patient Case: A 28-year-old patient is at 13 weeks of gestation (pregnancy). Tooth 36 is missing. Based on the following clinical scenario, what is the most appropriate implant-supported prosthetic treatment plan?
24. Patient Case: A 42-year-old patient is diagnosed with Hemophilia A. Teeth 16 and 17 are missing. Based on the following clinical scenario, what is the most appropriate implant-supported prosthetic treatment plan?
25. Patient Case: A 75-year-old patient with a stent (placed 3 years ago), diabetes, and hypertension. Medications include aspirin, clopidogrel, metformin, and beta-blockers. The maxilla is completely edentulous. Based on the following clinical scenario, what is the most appropriate implant-supported prosthetic treatment plan?
26. Patient Case: A 59-year-old patient presents with Sjögren’s syndrome and rheumatoid arthritis. Medications include methotrexate and alendronate. Teeth 24 and 25 are missing. Based on the following clinical scenario, what is the most appropriate implant-supported prosthetic treatment plan?
27. Patient Case: A 46-year-old patient with hypertension and Type 2 Diabetes Mellitus has an HbA1c of 8.7%. Teeth 25 and 27 are missing. Based on the following clinical scenario, what is the most appropriate implant-supported prosthetic treatment plan?
28. Patient Case: A 58-year-old patient is actively receiving chemotherapy and radiotherapy for thyroid cancer. Teeth 45, 46, and 47 are missing. Based on the following clinical scenario, what is the most appropriate implant-supported prosthetic treatment plan?
29. Patient Case: A 33-year-old patient is HIV positive. Teeth 14 and 17 are missing. Based on the following clinical scenario, what is the most appropriate implant-supported prosthetic treatment plan?
30. Patient Case: A 54-year-old patient with gout uses colchicine. Teeth 34 and 35 are missing. Based on the following clinical scenario, what is the most appropriate implant-supported prosthetic treatment plan?

**Part II: Anatomical Landmarks & Bone Deficiencies**

1. Patient Case: A 29-year-old patient with no systemic disease. There is a single tooth deficiency at 25 with a mesiodistal space of 4.0 mm. Based on the following clinical scenario, what is the most appropriate implant-supported prosthetic treatment plan?
2. Patient Case: A 49-year-old patient with no systemic disease. Teeth 15, 16, and 17 are missing. The residual bone height to the sinus floor is 3.0 mm. Based on the following clinical scenario, what is the most appropriate implant-supported prosthetic treatment plan?
3. Patient Case: A 61-year-old patient with no systemic disease. Teeth 35, 36, and 37 are missing. The distance to the mandibular canal is 2.3 mm, and the total bone height is 7.0 mm. Based on the following clinical scenario, what is the most appropriate implant-supported prosthetic treatment plan?
4. Patient Case: A 42-year-old patient with no systemic disease. Teeth 16 and 17 are missing. The sinus floor distance is 2.7 mm, and septa are present in the region. Based on the following clinical scenario, what is the most appropriate implant-supported prosthetic treatment plan?
5. Patient Case: A 39-year-old patient with no systemic disease. There is a single tooth deficiency at 47, with an observed increase in submandibular fossa depth. Based on the following clinical scenario, what is the most appropriate implant-supported prosthetic treatment plan?
6. Patient Case: A 35-year-old patient with no systemic disease. Tooth 16 is missing, and an oroantral fistula is detected. Based on the following clinical scenario, what is the most appropriate implant-supported prosthetic treatment plan?
7. Patient Case: A 48-year-old patient with no systemic disease. Teeth 11, 12, 21, and 22 are missing. The residual bone height to the nasal cavity floor is 0.8 mm. Based on the following clinical scenario, what is the most appropriate implant-supported prosthetic treatment plan?
8. Patient Case: A 37-year-old patient with no systemic disease. There is a single tooth deficiency at 46. The mental nerve distance to the anterior loop is 3.0 mm. Bone height is 11.0 mm and width is 4.0 mm. Based on the following clinical scenario, what is the most appropriate implant-supported prosthetic treatment plan?
9. Patient Case: A 31-year-old patient with no systemic disease. Teeth 14 and 15 are missing. The sinus floor distance is 6.0 mm. Buccolingual width is 5.0 mm and mesiodistal space is 9.0 mm. Based on the following clinical scenario, what is the most appropriate implant-supported prosthetic treatment plan?
10. Patient Case: A 44-year-old patient with no systemic disease. Teeth 11 and 21 were lost 2 years ago. The nasopalatine canal width is 6.7 mm. Based on the following clinical scenario, what is the most appropriate implant-supported prosthetic treatment plan?
11. Patient Case: A 57-year-old patient with no systemic disease. Teeth 31, 32, 41, and 42 are missing. The bone height is 8.1 mm, width is 2.7 mm, and distance to the lingual foramen is 1.3 mm (distance 1.9 mm). Based on the following clinical scenario, what is the most appropriate implant-supported prosthetic treatment plan?
12. Patient Case: A 52-year-old patient with no systemic disease. Tooth 33 is missing. Bone height is 11.5 mm, width is 2.3 mm, and lingual cortex thickness is 1.1 mm. Based on the following clinical scenario, what is the most appropriate implant-supported prosthetic treatment plan?
13. Patient Case: A 68-year-old patient with no systemic disease. Long-term complete mandibular edentulism. The buccolingual width in the anterior mandible is 3.6 mm, while the vertical height is sufficient. Based on the following clinical scenario, what is the most appropriate implant-supported prosthetic treatment plan?
14. Patient Case: A 33-year-old patient with no systemic disease. Tooth 22 was lost due to trauma, resulting in buccal bone loss. Based on the following clinical scenario, what is the most appropriate implant-supported prosthetic treatment plan?
15. Patient Case: A 47-year-old patient with no systemic disease. Teeth 41, 42, 31, and 32 are missing. Vertical bone height is sufficient, but the horizontal width is 3.8 mm. Based on the following clinical scenario, what is the most appropriate implant-supported prosthetic treatment plan?
16. Patient Case: A 61-year-old patient with no systemic disease. The patient is completely edentulous in both arches, with an interocclusal distance of 5.0 mm. Based on the following clinical scenario, what is the most appropriate implant-supported prosthetic treatment plan?
17. Patient Case: A 63-year-old patient with no systemic disease. Long-term edentulism in the 31–37 region. The residual bone height is 5.0 mm. Based on the following clinical scenario, what is the most appropriate implant-supported prosthetic treatment plan?
18. Patient Case: A 29-year-old patient with no systemic disease. Single tooth deficiency at 41. Buccolingual width is 2.7 mm and mesiodistal width is 3.0 mm. Based on the following clinical scenario, what is the most appropriate implant-supported prosthetic treatment plan?
19. Patient Case: A 55-year-old patient with no systemic disease. Edentulism in the 31–33 and 41–43 regions. Bone height is 5.0 mm on the left and 7.0 mm on the right. Based on the following clinical scenario, what is the most appropriate implant-supported prosthetic treatment plan?
20. Patient Case: A 68-year-old patient with no systemic disease. The maxilla is completely edentulous, with severe posterior bone loss (<3.0 mm) and sinus pneumatization. Based on the following clinical scenario, what is the most appropriate implant-supported prosthetic treatment plan?
21. Patient Case: A 53-year-old patient with no systemic disease. Teeth 16, 17, 27, 37, and 47 are missing. In the 16–17 region, the sinus distance is 8.0 mm and bone width is sufficient. Based on the following clinical scenario, what is the most appropriate implant-supported prosthetic treatment plan?
22. Patient Case: A 27-year-old patient with no systemic disease. A lesion originating from tooth 37 has caused bone resorption up to the middle third of the root. Based on the following clinical scenario, what is the most appropriate implant-supported prosthetic treatment plan?
23. Patient Case: A 55-year-old patient with no systemic disease. The mandible presents a knife-edge ridge morphology. Based on the following clinical scenario, what is the most appropriate implant-supported prosthetic treatment plan?
24. Patient Case: A 35-year-old patient with no systemic disease. Teeth 12 and 22 are congenitally missing. Bone height is insufficient both vertically and horizontally. Based on the following clinical scenario, what is the most appropriate implant-supported prosthetic treatment plan?
25. Patient Case: A 66-year-old patient with no systemic disease. Bone defects are present due to long-term prosthesis use. Based on the following clinical scenario, what is the most appropriate implant-supported prosthetic treatment plan?
26. Patient Case: A 44-year-old patient with no systemic disease. Tooth 45 is missing, with observed buccal bone loss. Based on the following clinical scenario, what is the most appropriate implant-supported prosthetic treatment plan?
27. Patient Case: A 63-year-old patient with no systemic disease. Teeth 31, 32, 41, and 42 are missing. Bone height is 9.0 mm and thickness is 2.3 mm. Based on the following clinical scenario, what is the most appropriate implant-supported prosthetic treatment plan?
28. Patient Case: A 67-year-old patient with no systemic disease. Missing tooth 33 and 35. The mental nerve distance to the crest is 7.0 mm, and a knife-edge ridge is present. Based on the following clinical scenario, what is the most appropriate implant-supported prosthetic treatment plan?
29. Patient Case: A 56-year-old patient with no systemic disease. Teeth 13 and 12 are missing. Crest height is 8.9 mm and width is 2.7 mm. Based on the following clinical scenario, what is the most appropriate implant-supported prosthetic treatment plan?
30. Patient Case: A 61-year-old patient with no systemic disease. Long-term history of hypertension medication use. Sinus floor distance is 5.6 mm (16–17 region) and 3.7 mm (24–26 region). The mandibular canal to crest distance is 11.7 mm (35–37 region) and 9.3 mm (34–37 region). Based on the following clinical scenario, what is the most appropriate implant-supported prosthetic treatment plan?
